# Degradable Acrylate-Telechelic Copolymers with Disulfide and β-Thioester Linkages by Simultaneous Thiol Oxidation and Thiol-Ene Michael Addition Click Reactions with the Same Base Catalyst

**DOI:** 10.3390/polym18182211

**Published:** 2026-09-11

**Authors:** Ákos Szabó, Aiman Aitkazina, Györgyi Szarka, Dóra Fecske, Anna Petróczy, Béla Iván

**Affiliations:** 1 Polymer Chemistry and Physics Research Group, Institute of Materials and Environmental Chemistry, HUN-REN Research Centre for Natural Sciences, Magyar Tudósok krt. 2, H-1117 Budapest, Hungary; 2 Hevesy György Doctoral School of Chemistry, Institute of Chemistry, Faculty of Science, Eötvös Loránd University, Pázmány Péter Sétány 1/A, H-1117 Budapest, Hungary

**Keywords:** sulfur-containing copolymer, simultaneous oxidative thiol–thiol coupling and thiol-ene click reaction, disulfide and β-thioester linkages, 3,6-dioxa-1,8-octane-dithiol (DODT), poly(ethylene glycol) diacrylate (PEGDA), 1,6-hexanediol diacrylate (HDODA), *N*,*N*,*N′*,*N″*,*N″*-pentamethyldiethylenetriamine (PMDETA) base catalyst, acrylate-telechelic P(DODT-*co*-diacrylate) copolymers, low *T*_g_ copolymers, chain-breaking degradation by disulfide–thiol exchange, hydrolysis by strong base

## Abstract

This study reports on a new one-pot copolymerization process by simultaneous oxidative disulfide and β-thioester formation by reacting bifunctional monomers, 3,6-dioxa-1,8-octane-dithiol (DODT) with diacrylates, poly(ethylene glycol) diacrylate (PEGDA) and 1,6-hexanediol diacrylate (HDODA), in the presence of *N*,*N*,*N′*,*N″*,*N″*-pentamethyldiethylenetriamine (PMDETA), as the same base catalyst for both reactions, in air at room temperature with short reaction times. The resulting random copolymers consist of disulfide linkages between DODTs and β-thioester units formed by thiol-ene Michael addition click reaction. With a stoichiometric DODT/diacrylate feed ratio, diacrylate-telechelic copolymers are obtained in a single-step polymerization reaction. The *T*_g_s of the P(DODT-*co*-PEGDA) copolymers are nearly constant at around −53 °C, while it decreases with increasing HDODA content in the P(DODT-*co*-HDODA) copolymers. Reductive degradation with thiols, such as 2-mercaptoethanol and dithiothreitol, led to chain scission via the disulfide–thiol exchange reaction. Treatment with NaOH solution resulted in further degradation by hydrolysis of the β-thioester units. These results indicate that these novel copolymers are fully degradable under mild conditions. This new process, applying simultaneous thiol oxidation and thiol-ene reactions, enables to prepare a large variety of sulfur-containing end-functional degradable copolymers useful for a broad range of advanced application possibilities.

## 1. Introduction

Sulfur-containing polymers have gained significant interest in recent years due to the versatile properties and advanced application possibilities of such materials, ranging from recyclability to energy storage devices, e.g., Li-sulfur batteries, biomaterials, drug delivery matrices, self-healing constructs, metal-ion adsorbents, etc. (see, e.g., Refs. [1,2,3,4,5,6,7,8,9,10,11] and references therein). Undoubtedly, among such materials, macromolecules with in-chain or pendant disulfide linkages belong to one of the most widely investigated classes of polymers nowadays [8,9,10,11,12,13,14,15,16,17,18,19,20,21,22,23,24,25,26,27,28,29,30,31,32,33,34,35,36,37,38]. This is related to the unique dynamic redox property of the disulfides, that is, cleavage by reduction and disulfide formation by oxidation of the resulting thiols, which can be purposefully utilized in various fields. Polymers by disulfide units can be obtained, for instance, by ring-opening polymerization of dithiolanes [11,12,13,14,15,16,17,18], polymerization of disulfide-containing monomers [19,20,21,22,23,24,25], thiol–disulfide exchange [26] or oxidative coupling of bifunctional or multifunctional thiols [27,28,29,30,31,32,33,34,35,36,37,38]. An interesting oxidative coupling process of dithiols, such as 3,6-dioxa-1,8-octane-dithiol (DODT), thiol-telechelic polyisobutylene and alkane-dithiols, by hydrogen peroxide in air via catalysis with a base for the formation of disulfide-containing polymers with unique properties was reported by Puskas et al. [32,33,34,35,36,37]. These authors called this reaction radical ring-opening redox polymerization (R3P), which is claimed to result in macrocycles and to fulfill the green chemistry criteria as well [32,33,34].

Polymers formed by thiol-ene click reactions belong to another widely investigated class of sulfur-containing macromolecules. Although the addition of a thiol to an olefinic double bond was discovered in the early years of the 20th century [39], this reaction has been in the focus of scientific interest in polymer research and development only in the last two decades [40,41,42,43,44,45,46,47,48,49,50,51,52,53,54,55,56,57,58,59,60,61,62]. There are two major processes to obtain polymers by thiol-ene reactions, i.e., radical addition of thiyl radicals, formed by thermal initiation or photoinitiation, to double-bond-containing molecules [40,41,42,43] or by thiol-ene Michael addition [44,45,46,47,48,49,50,51,52,53,54,55,56,57,58,59,60,61,62]. The thiol-ene Michael addition reaction can be catalyzed by a nucleophile, usually by a base, such as amines, leading to a protonated base and thiolate anion, which react with the olefin, and the formed carbanion is neutralized by capturing the proton from the protonated base [62]. By using bifunctional or multifunctional thiols and acrylates, a variety of poly(β-thioester)s or polymers with β-thioester moieties have been prepared by the thiol-ene Michael addition click reaction [44,45,46,47,48,49,50,51,52,53,54,55,56,57,58,59,60,61]. Junkers et al. [44,45,46,47] reported on the synthesis of poly(β-thioester)s with this click polymerization process and demonstrated the degradability of these polymers via hydrolysis with strong bases and also under biologically relevant conditions [45,46]. Others accomplished the synthesis of various β-thioester containing polymers with a broad range of application possibilities, such as solid polymer electrolytes, polymer–peptide conjugates, herbicide delivery polymers, hyperbranched polymers, noble metal recovery materials, thermoresponsive polymers, hydrogels, reprocessable elastomers, polymers with high encapsulation stability, etc. [48,49,50,51,52,53,54,55,56,57,58,59,60]. Kohsaka and coworkers [61] carried out step-growth tandem polymerization of dithiols and α-(halomethyl)acrylates with the involvement of the thiol-ene Michael addition reaction in this process. These examples indicate well the versatility of the thiol-ene Michael addition click reaction for obtaining various novel macromolecular assemblies with unique structure, properties and application possibilities.

In this study, inspired by the common feature of the oxidative coupling of thiols with hydrogen peroxide as the oxidant and the thiol-ene Michael addition click reactions, that is, by using the same catalyst, i.e., a base, we report on a new process and the resulting novel copolymers containing both disulfide and β-thioester units, formed simultaneously in one reaction medium. We attempted the synthesis of such a new class of copolymers by the competitive oxidative coupling of a dithiol and the thiol-ene reaction by adding diacrylate comonomers, poly(ethylene glycol) diacrylate (PEGDA) and 1,6-hexanediol diacrylate (HDODA), to the oxidative polymerization of DODT with H_2_O_2_ in the presence of a base in open air and under energy-saving room-temperature conditions. Surprisingly, this easy-to-use process led to copolymers containing not only degradable disulfide and β-thioester units, but to acrylate-terminated polymers as well under proper conditions without the need for any additional end-functionalization process. This robust new synthetic route, which combines two simultaneous reactions with the involvement of a dithiol and a diacrylate, and the resulting functional degradable copolymers are expected to open new ways for the preparation of a variety of novel macromolecular assemblies.

## 2. Materials and Methods

### 2.1. Materials

3,6-Dioxa-1,8-octane-dithiol (DODT) and *N*,*N*,*N′*,*N″*,*N″*-pentamethyldiethylenetriamine (PMDETA) were purchased from TCI (Zwijndrecht, Belgium), 1,6-hexanediol diacrylate (HDODA) from Polysciences (Hirschberg, Germany), tetrahydrofuran (THF) and methanol from VWR International (Debrecen, Hungary), whereas poly(ethylene glycol) diacrylate (*M*_n_ = 250 g/mol) (PEGDA), 30 wt% aqueous solution of H_2_O_2_, 2-mercaptoethanol (2-ME) and dithiothreitol (DTT) were purchased from Sigma Aldrich (Steinheim, Germany). PEGDA and HDODA were purified by passing through a column filled with basic Al_2_O_3_. Other reagents were used as received.

### 2.2. Synthesis Procedures

#### 2.2.1. Preparation of P(DODT-*co*-PEGDA) and P(DODT-*co*-HDODA) Copolymers

A representative copolymerization of DODT and PEGDA in a 1:1 molar ratio was carried out in a round-bottom flask open to air by adding first 0.25 mL (1.54 mmol) of DODT dissolved in 3.0 mL THF. Subsequently, 0.21 mL (1.01 mmol) PMDETA (1 equivalent amine to the thiol groups) and 0.35 mL (1.55 mmol) PEGDA were added to the reaction mixture followed by the immediate addition of 0.75 mL 6.5 wt% diluted aqueous solution of H_2_O_2_ (1.47 mmol, withdrawn from the mixture of 1.0 mL 30 wt% H_2_O_2_ solution and 4.0 mL distilled water). After 15 min of reaction time, methanol was added to the opaque solution in which the obtained polymer was precipitated, followed by decanting of the solvent and drying of the resulting polymer to constant weight under vacuum at room temperature.

For the scaled-up synthesis of the P(DODT-*co*-PEGDA) copolymer, DODT (16.6 mL, 0.1 mol), PEGDA (23.3 mL, 0.1 mol), and PMDETA (14.0 mL, 0.067 mol) were dissolved in distilled THF (200 mL), followed by the immediate addition of hydrogen peroxide (10 mL, 6.5 wt%). The reaction mixture was stirred without external heating or cooling at ambient temperature for 10 min. After completion of the reaction, the copolymer was precipitated by addition of a large excess of methanol. The resulting milky suspension was left to sediment, followed by decanting of the solvent and drying of the resulting polymer to constant weight under vacuum at room temperature.

#### 2.2.2. Experiments with Sequential Addition of the Reactants

Experiments with sequential addition of the reactants were performed by charging the components in open reactors at room temperature at predetermined time intervals. First, to the solution of 0.5 mL (3.07 mmol) DODT, 0.43 mL (2.06 mmol) PMDETA, 6.0 mL THF and 0.69 mL (3.06 mmol) PEGDA, 1.57 mL of a 6.5 wt% diluted aqueous solution of H_2_O_2_ (3.07 mmol) was added. After 15 min of reaction time, the half-volume of the reaction mixture was withdrawn and precipitated in methanol (sample *s1-1*). Subsequently, the solution of 0.25 mL (1.54 mmol) DODT, 0.22 mL (1.05 mmol) PMDETA, 3.0 mL THF and 0.35 mL (1.55 mmol) PEGDA were added, followed by charging of 0.78 mL of a 6.5 wt% aqueous solution of H_2_O_2_ (1.53 mmol). After another 15 min of reaction time, half of the reaction mixture was also withdrawn and precipitated in methanol (sample *s1-2*) followed by the addition of the same monomer and oxidant solution in the remaining reaction mixture as mentioned above. After a subsequent 15 min of reaction time, the experiment was stopped by the precipitation of the formed polymer in methanol (sample *s1-3*).

The experiment with sequential DODT and oxidant addition was performed by first carrying out the reaction with charging 1.57 mL of 6.5 wt% aqueous solution of H_2_O_2_ (3.07 mmol) to the solution of 0.5 mL (3.07 mmol) DODT, 0.43 mL (2.06 mmol) PMDETA, 6.0 mL THF and 0.69 mL (3.06 mmol) PEGDA. After 15 min of reaction time, half of the volume of the reaction mixture was withdrawn and precipitated in methanol (sample *s2-1*). Subsequently, the solution of 0.25 mL (1.54 mmol) DODT, 0.22 mL (1.05 mmol) PMDETA and 3.0 mL THF were added, followed by charging of 0.78 mL of 6.5 wt% diluted aqueous solution of H_2_O_2_ (1.53 mmol). After another 15 min of reaction time, half of the reaction mixture was also withdrawn and precipitated in methanol (sample *s2-2*), followed by the addition of the same monomer and oxidant solution as mentioned above. After a subsequent 15 min of reaction time, the experiment was stopped by precipitation of the formed polymer in methanol (sample *s2-3*).

#### 2.2.3. Reduction of the Disulfide Units in P(DODT-*co*-PEGDA) and P(DODT-*co*-HDODA) Copolymers

For the reduction, that is, the scission of the disulfide linkages, 60 mg of the given copolymer was dissolved in 3.0 mL THF and 0.053 mL (0.76 mmol) 2-mercaptoethanol was added to the reaction mixture. After stirring for 17 h at room temperature, methanol was added to the solution in which the product was precipitated, and then dried under vacuum at room temperature.

Further reductive degradation studies of the P(DODT-*co*-PEGDA) copolymer were performed using dithiothreitol (DTT), followed by the addition of NaOH solution for hydrolysis of the β-thioester linkages. For these experiments, P(DODT-*co*-PEGDA) (0.40 g, *M*_n_ = 4500 g/mol) was dissolved in acetonitrile (5 mL), followed by the addition of 0.40 g DTT (2.60 mmol). The reaction mixture was stirred at room temperature for 48 h. Subsequently, the resulting solution was divided into two equal portions. The first portion was concentrated under reduced pressure, dried overnight under vacuum at room temperature, washed with diethyl ether, and dried again under vacuum for subsequent characterization. The second portion was subjected to alkaline hydrolysis by adding 2 mL of aqueous NaOH solution (1 M) under continuous nitrogen bubbling. After stirring overnight at room temperature, chloroform was added to extract the degradation products. The organic phase was separated, dried over anhydrous MgSO_4_, concentrated under reduced pressure, and dried under vacuum.

### 2.3. Characterization Methods

The ^1^H NMR spectra of the polymers were recorded on a Varian 500 MHz spectrometer (Varian Inova, Palo Alto, CA, USA) in CDCl_3_ at room temperature.

Gel permeation chromatography (GPC) of the synthesized polymers was carried out with an instrument equipped with two Styragel HR columns (HR1 and HR4), Waters 515 HPLC pump, Waters 717 Autosampler, Jetstream Column Thermostat (Waters, Milford, MA, USA) and Agilent 1260 Infinity refractive index detector (Agilent, Santa Clara, CA, USA), applying tetrahydrofuran as an eluent with 0.3 mL/min flow rate at 35 °C. The molecular weight data were obtained by evaluation of the chromatograms on the basis of calibration made with poly(ethylene glycol) standards of narrow molecular weight distribution.

The solubility of the copolymers was evaluated by mixing approximately 50–100 mg of copolymer with 1 mL of the respective solvent at room temperature, followed by manual agitation. No heating or sonication was applied. Samples forming a homogeneous solution without visible undissolved polymer were classified as soluble, whereas samples containing persistent undissolved polymer were classified as insoluble.

The FTIR-ATR measurements were performed on a Bruker Tensor 27 FTIR instrument (Bruker Optics, Ettlingen, Germany). The spectra were obtained by using 32 scans with a resolution of 2 cm^−1^ in the range of 400–4000 cm^−1^.

Differential scanning calorimetry (DSC) measurements were performed on a Mettler TG50 equipment (Mettler Toledo, Leicester, UK) in the −100 °C to +200 °C temperature range with 10 °C/min heating rate. The second heating curves were evaluated, and the inflection points of the DSC thermograms were recorded as glass transition temperatures.

## 3. Results and Discussion

### 3.1. The Synthesis of P(DODT-co-PEGDA) Copolymers with Disulfide and β-Thioester Linkages via Two Simultaneous Click Reactions

**Scheme 1:**
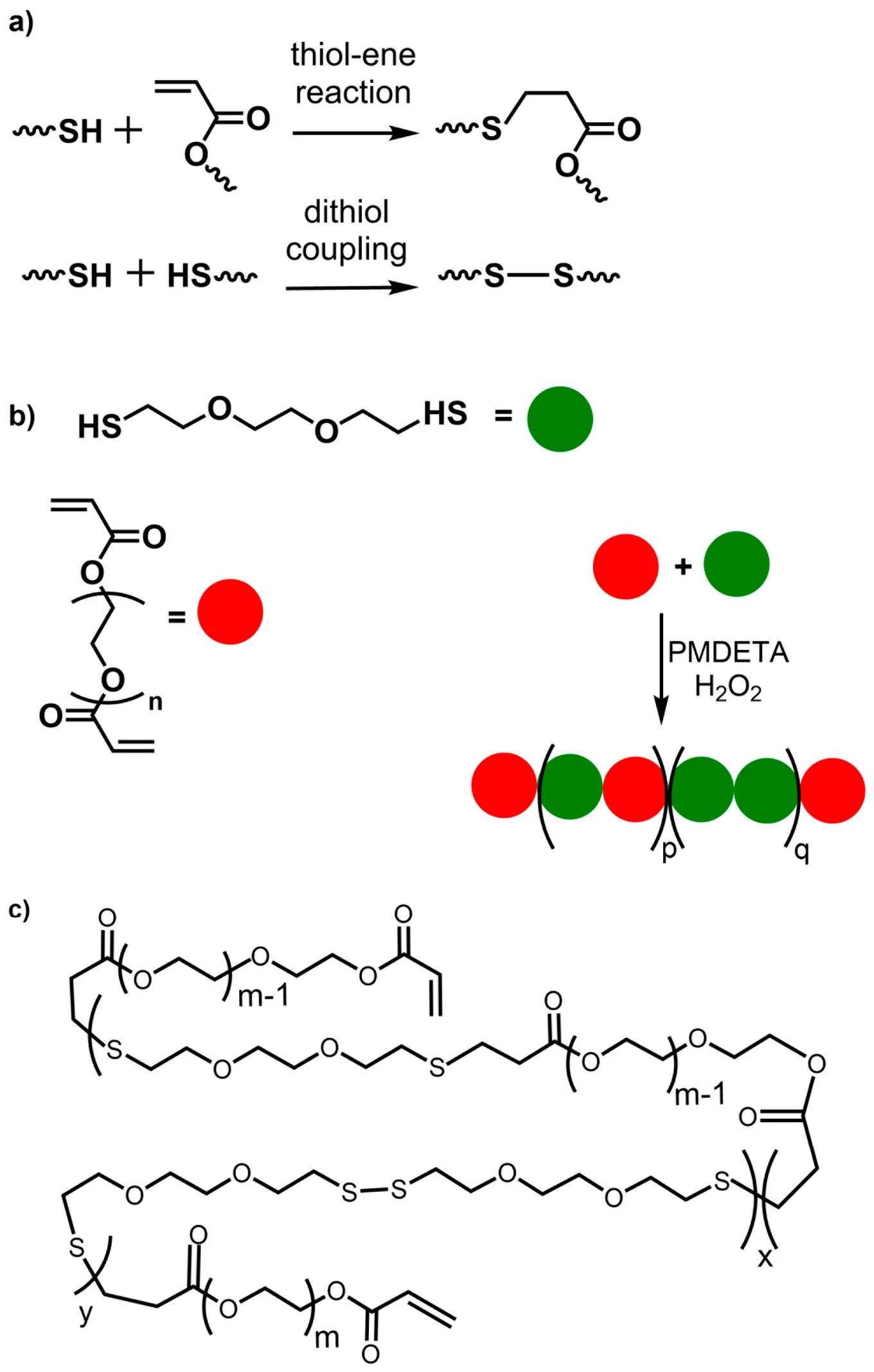
The formation of P(DODT-*co*-PEGDA) copolymers: (**a**) the two simultaneous reactions by thiol-ene addition and oxidative coupling of thiols, (**b**) the scheme of the copolymerization of DODT and PEGDA, and (**c**) the structure of the formed copolymer.

A thiol is known to form thiolate by reacting with amine bases even under mild conditions [62]. In the simultaneous presence of an oxidant, like H_2_O_2_, and an acrylate, two processes can take place. The oxidation of the thiolate with H_2_O_2_ leads to thiyl radicals, the combination of which results in a disulfide, as proposed for the oxidative polymerization of DODT [32,33,34,35,36,37], while the Michael addition of the thiolate to the double bond of the acrylate yields a β-thioester [62] as displayed in Scheme 1a. To investigate whether the thiolate can take part simultaneously in both the thiol-ene Michael addition and the disulfide formation by the oxidation reaction, the polymerization of a dithiol, DODT, was performed in the presence of a bifunctional acrylate comonomer, poly(ethylene glycol) diacrylate (PEGDA) (Table 1). As shown in Table 1, polymers with yields ranging from 40 to 70% were formed in this process.

**Figure 1:**
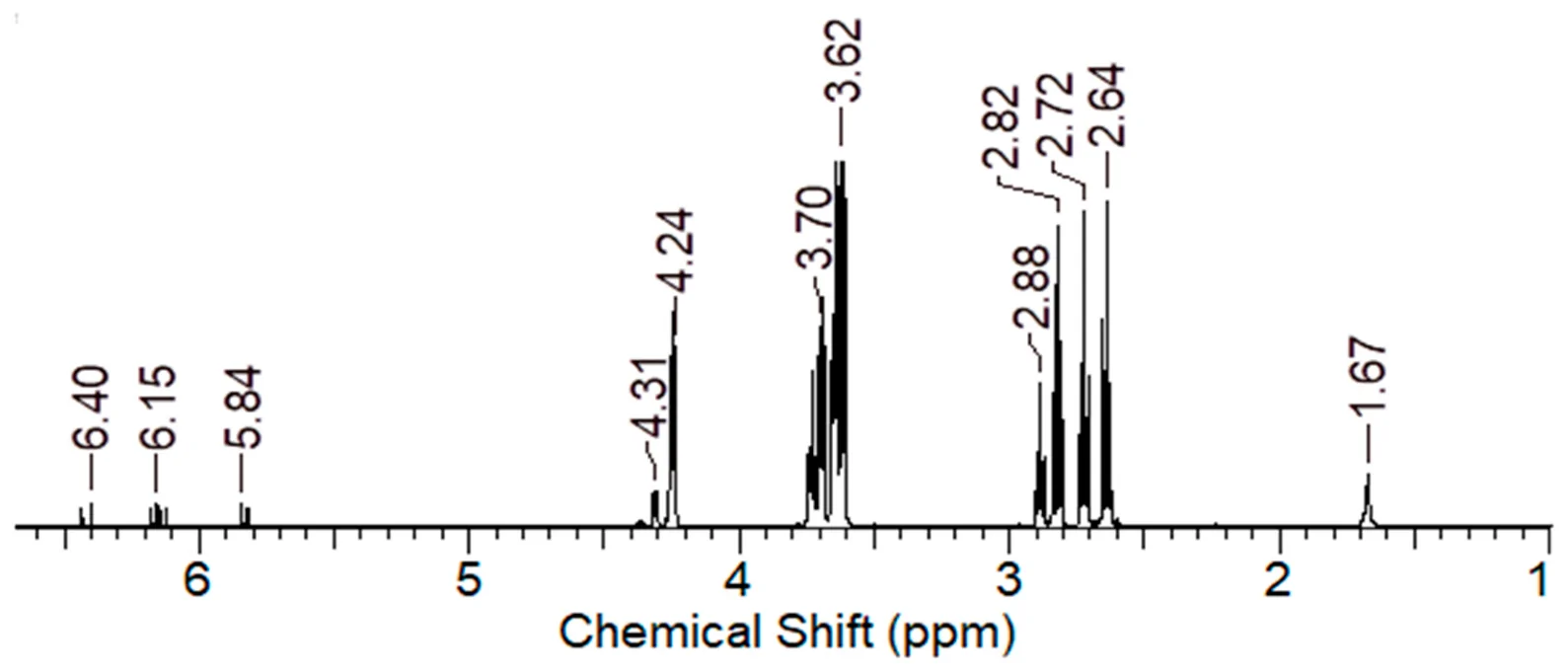
The ^1^H NMR spectrum of the P(DODT-*co*-PEGDA) copolymer obtained in the experiment *c1*-50 (50 mol% DODT and 50 mol% of PEGDA in the feed).

Figure 1 shows the ^1^H NMR spectrum of the polymeric product obtained with 50:50 molar ratio of DODT and PEGDA in the feed (*c1*-50). The absence of the thiol (–SH) triplet peak at 1.58 ppm indicates that there are no –SH chain end groups in the formed macromolecules [32]. However, it can be concluded that unreacted terminal acrylate groups, the signals of whose olefinic protons can be found at 6.40, 6.15 and 5.84 ppm, are present in these polymer chains. In addition to the triplet peak of the methylene protons adjacent to the disulfide bonds at 2.88 ppm, three different signals can be found at 2.82, 2.72 and 2.64 ppm. Since all of these three peaks are triplets with the same integrated area, they can be assigned to the methylene protons of the ~–CH_2_–S–CH_2_–CH_2_–CO–O–~ β-thioester sequences in the chains, formed by thiol-ene Michael addition between DODT and PEGDA. This is in accordance with the presence of the two triplets at 4.24 and 4.31 ppm, the former of which has the same integrated area as the methylenes adjacent to the C-S-C sulfur, while the latter has an integrated area value twice of the peaks of the olefinic protons. Therefore, these two triplets at 4.24 and 4.31 ppm can be assigned as the methylene groups adjacent to the ester groups in the saturated and unsaturated acrylate groups, respectively. The multiplets at 3.62 and 3.70 ppm belong to the ethylene glycol groups both in the PEGDA and DODT units, and their total integrated area is in good accordance with the calculated amount of the incorporated PEGDA and DODT units determined on the basis of the aforementioned assignations. All these indicate that the reaction shown in Scheme 1b can be proposed for the formation of P(DODT-*co*-PEGDA) copolymers, that is, the thiol-ene coupling reaction occurs between the DODT and PEGDA simultaneously with the oxidative coupling of DODT, yielding random copolymers with both disulfide and β-thioester units in the chain. Thus, this structural analysis clearly indicates that a copolymer with terminal acrylate groups is formed with 50:50 molar ratio of DODT/PEGDA in the feed. The structure of this copolymer is displayed in Scheme 1c. From the ^1^H NMR spectra, the DODT and PEGDA contents in the copolymers can also be estimated (Table 1). Although somewhat higher DODT content is found in the copolymers than in the feed, which is due to the consumption of DODT in the disulfide formation in addition to the thiol-ene reaction, the composition of these novel copolymers can be well controlled in a broad composition range by the proper variation in the DODT/PEGDA feed ratio.

**Figure 2:**
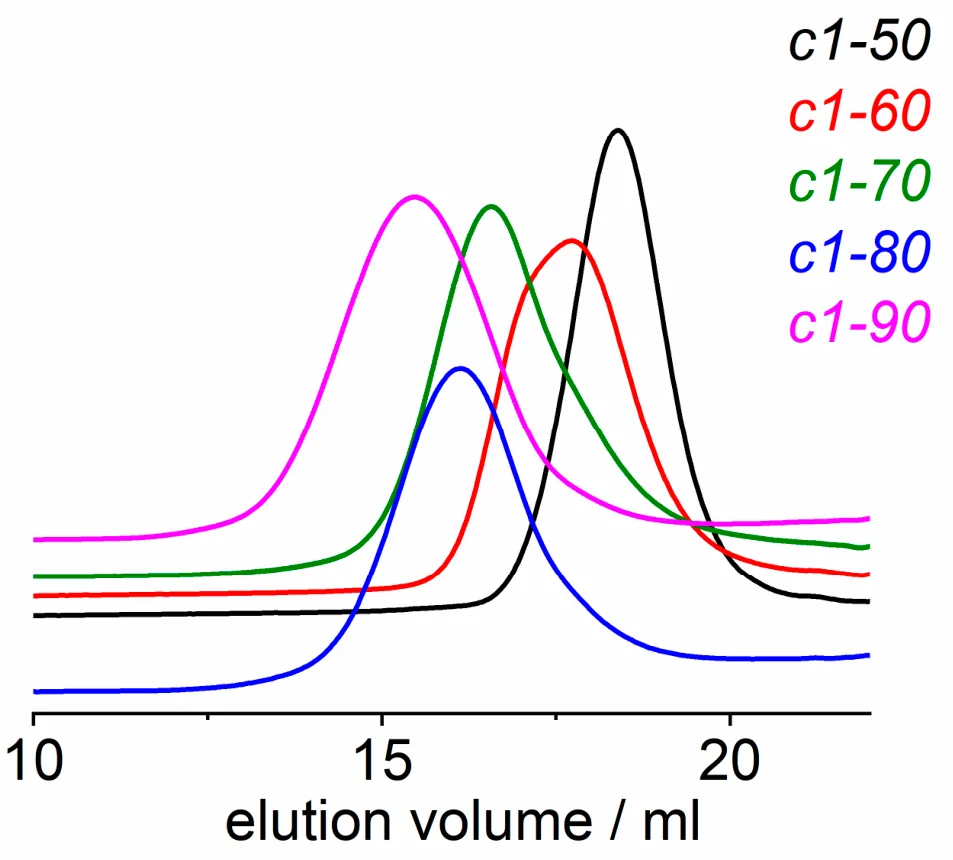
The GPC chromatograms of the copolymers obtained in the *c1* experimental series (see Table 1 for sample identification).

As presented in Figure 2, gel permeation chromatography analysis also proves the formation of uniform polymer products, because monomodal peaks can be observed in all cases. As the data clearly shows in Table 1, copolymers with relatively narrow molecular weight distributions, with dispersity values in the range of 1.3–2.3 range, are obtained, and increasing the DODT content in the feed results in molecular weight increase (Table 1) in line with the shift of the GPC chromatograms toward lower elution volumes as displayed in Figure 2. The molecular weight distributions (MWDs) obtained by converting the GPC curves on the basis of the calibration made by linear poly(ethylene glycol) homopolymer standards are displayed in Figure S1 in the Supplementary Materials. However, due to the differences in the hydrodynamic volume versus molecular weight correlation between the standards and the investigated copolymers, these data, including the data in Table 1 as well, can be taken into account as relative rather than absolute MW values for qualitative evaluation of the trends in changes in the MWs.

**Scheme 2:**
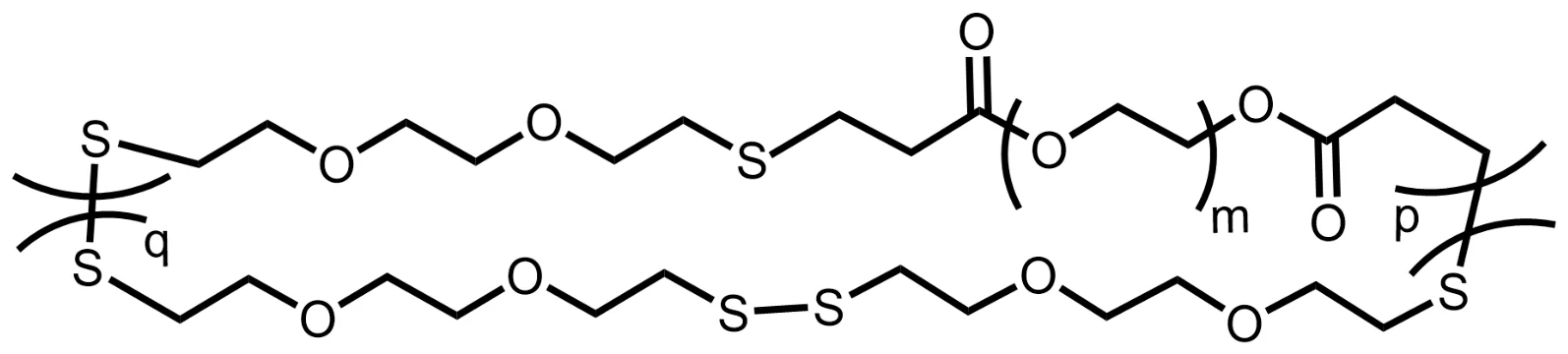
The structure of the P(DODT-*co*-PEGDA) copolymer macrocycles.

The number average molecular weights (*M*_n_) can also be determined on the basis of the integral values of the signals of the ^1^H NMR spectra of the P(DODT-*co*-PEGDA) copolymers when the signals of the terminal acrylate groups are visible in the spectra, such as in the case of the *c1*-50 sample, that is, when stoichiometric amounts of DODT and PEGDA are reacted. This led to *M*_n_ of 4.5 kg/mol, which is relatively close to the *M*_n_ value estimated by the evaluation of the GPC curve. However, when the DODT is in excess to PEGDA, like in the case of the *c1*-60 sample, the *M*_n_ determination on the basis of integration of the acrylate double bond signals leads to higher-than-expected *M*_n_ value. The presence of acrylate double bonds cannot be detected in the ^1^H NMR spectra of samples prepared with higher DODT/PEGDA ratios (samples *c1*-70, *c1*-80 and *c1*-90). These findings are in agreement with the reported macrocycle formation between oxidized DODT by combination of the thiyl radicals [32,33,34], and the macrocyclic copolymer chains do not have acrylate terminal groups as shown in Scheme 2. These findings allow us to conclude that, while macrocycles are obtained with higher than one DODT/PEGDA ratios, acrylate-telechelic P(DODT-*co*-PEGDA) copolymers are formed with stoichiometric DODT-PEGDA feed ratio in the course of simultaneous oxidative disulfide formation and thiol-ene Michael addition catalyzed by PMDETA as a base catalyst.

**Figure 3:**
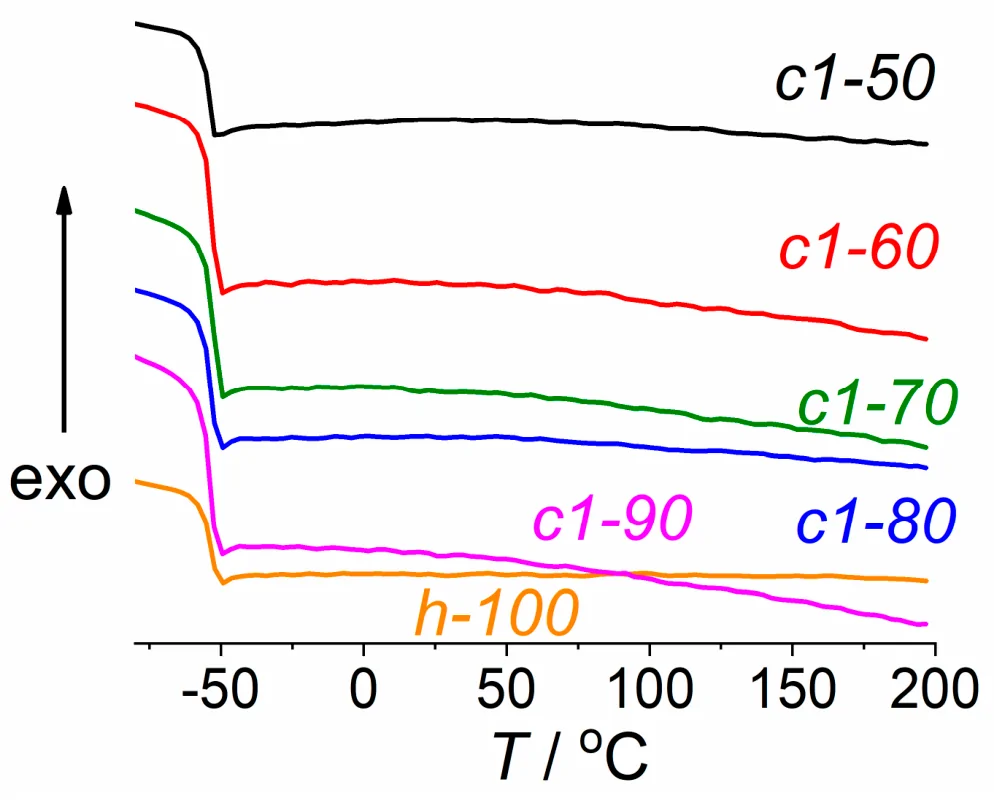
The differential scanning calorimetry curves of the P(DODT-*co*-PEGDA) copolymers and the PDODT homopolymer (*h*-100) obtained in the c1 experimental series (see Table 1).

**Figure 4:**
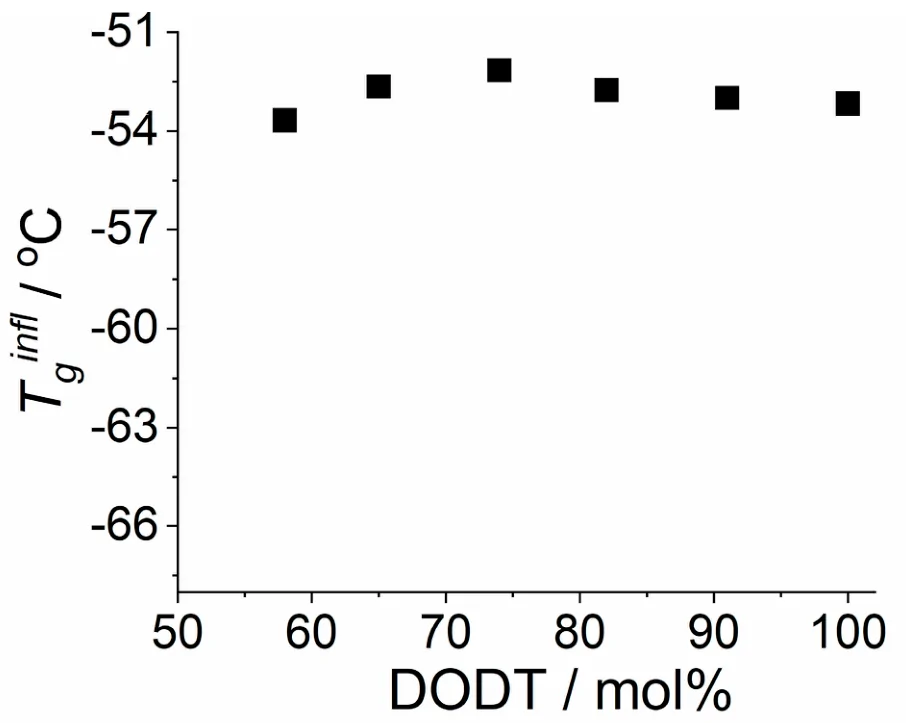
The glass transition temperatures as a function of the DODT content of the P(DODT-*co*-PEGDA) copolymers obtained in the c1 experimental series and the PDODT homopolymer (*h*-100) (see Table 1).

Differential scanning calorimetry (DSC) measurements were also performed on the obtained copolymer samples. The DSC curves indicate the presence of only one glass transition in the −100–+200 °C temperature range (Figure 3). The glass transition temperatures of the P(DODT-*co*-PEGDA) copolymers, however, do not change with the increasing DODT content in the copolymers (Figure 4), which can be explained by the presence of ethylene oxide units in both the PEGDA and DODT monomer units.

The solubility of the P(DODT-*co*-PEGDA) copolymers was tested in a series of solvents from highly polar water to low-polarity compounds, like *n*-hexane and diethyl ether. As shown in Table 2, unexpectedly, the copolymers are not soluble in polar solvents, such as water, methanol and ethanol, although both pure comonomers, having ethylene glycol units, are soluble in such solvents, like low-molecular-weight alcohols. This finding can be explained by the formation of the β-thioester and the nonpolar disulfide linkages in these copolymers. As expected, nonpolar solvents, such as *n*-hexane, diethyl ether, are also non-solvents for the P(DODT-*co*-PEGDA) copolymers. Polar organic solvents, which are known as good solvents for polyesters, like acetone, THF, methyl ethyl ketone, acetonitrile, DMF, etc., proved to be good solvents for these novel copolymers.

### 3.2. P(DODT-co-HDODA) Copolymers by Simultaneous Disulfide and β-Thioester Formation

**Figure 5:**
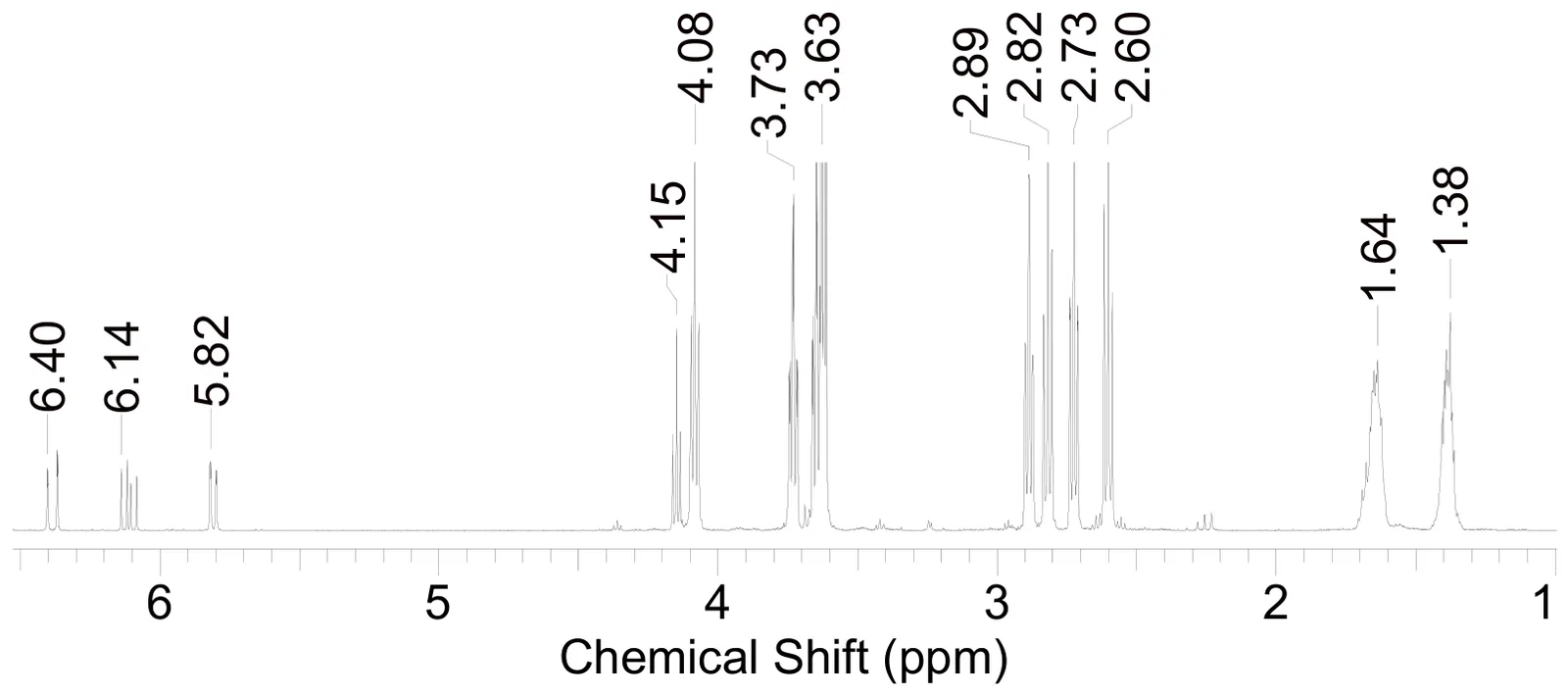
The ^1^H NMR spectrum of the P(DODT-*co*-HDODA) copolymer obtained in the experiment *c2*-50 (50 mol% of DODT and 50 mol% of HDODA in the feed).

The copolymerization of DODT was also carried out with another acrylate comonomer, 1,6-hexanediol diacrylate (HDODA) in the presence of H_2_O_2_ oxidant and the PMDETA amine base. Figure 5 shows the ^1^H NMR spectrum of the copolymer obtained by using 1:1 molar ratio of DODT and HDODA under conditions identical to that of the preparation of the P(DODT-*co*-PEGDA) copolymers. This spectrum clearly indicates the formation of P(DODT-*co*-HDODA) with acrylate termini and a structure similar to that displayed for the P(DODT-*co*-PEGDA) copolymers with disulfide and β-thioester linkages as shown in Scheme 1c, with the difference that HDODA units replace the PEGDA segments. As the data in Table 3 indicate, the molar ratio of DODT incorporated in the obtained copolymers, determined on the basis of the ^1^H NMR spectra, is higher than in the feed or nearly equal (as in samples *c2*-80 and *c2*-90). It is also noteworthy to mention that the incorporation of DODT in relative excess decreases systematically with its increasing feed ratio, that is, from 11 mol% for the *c2*-50 sample (61 mol% in the sample compared to 50 mol% in the feed) to practically zero in the *c2*-80 and *c2*-90 samples.

**Figure 6:**
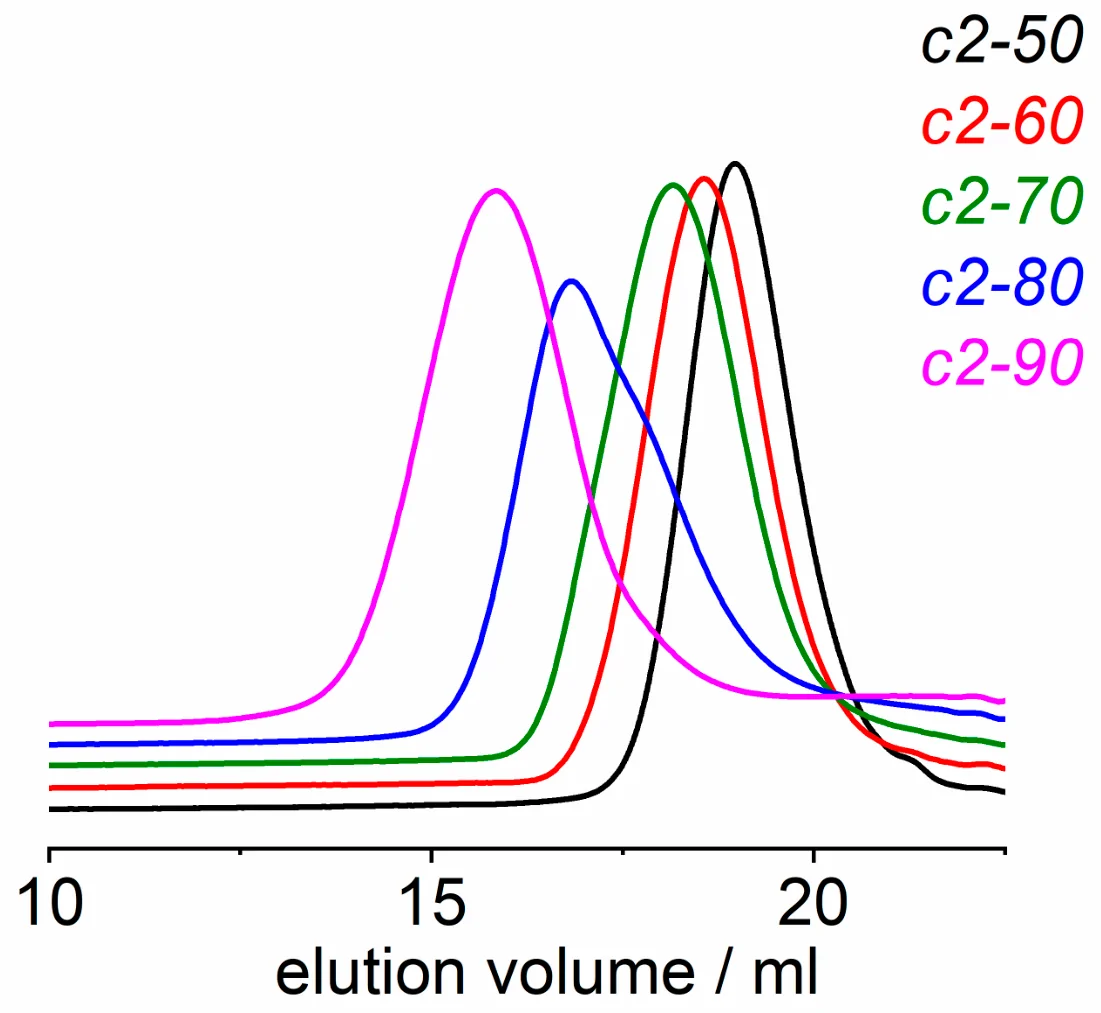
The gel permeation chromatograms of the P(DODT-*co*-HDODA) copolymers obtained in the experimental series of *c2* (see Table 3 for sample identification).

The GPC chromatograms and the corresponding MWDs of the P(DODT-*co*-HDODA) copolymers are displayed in Figure 6 and Figure S2, respectively. Similar to the P(DODT-*co*-PEGDA) copolymers, the GPC curves clearly indicate that copolymers with monomodal distributions are formed in the reaction of DODT with HDODA as well, on the one hand. On the other hand, as these chromatograms and the molecular weight data in Table 3 show, increasing the DODT/HDODA ratio in the feed leads to an increase in the molecular weights of the formed copolymers. The *M*_n_ determined on the basis of the integration values of the ^1^H NMR signals in comparison to the integration value of the acrylate double bond signals indicates the same trend, that is, the molecular weight of the copolymers increases with the increasing DODT content in the feed. This is due to the higher probability of the chain–chain coupling via disulfide formation, which is reflected by the fact that the molecular weight distributions broaden with increasing DODT content. Because thiol end groups cannot be detected in the NMR spectra, it can be concluded that the chains with higher DODT contents may form macrocycles, like in the case of the P(DODT-*co*-PEGDA) copolymers.

**Figure 7:**
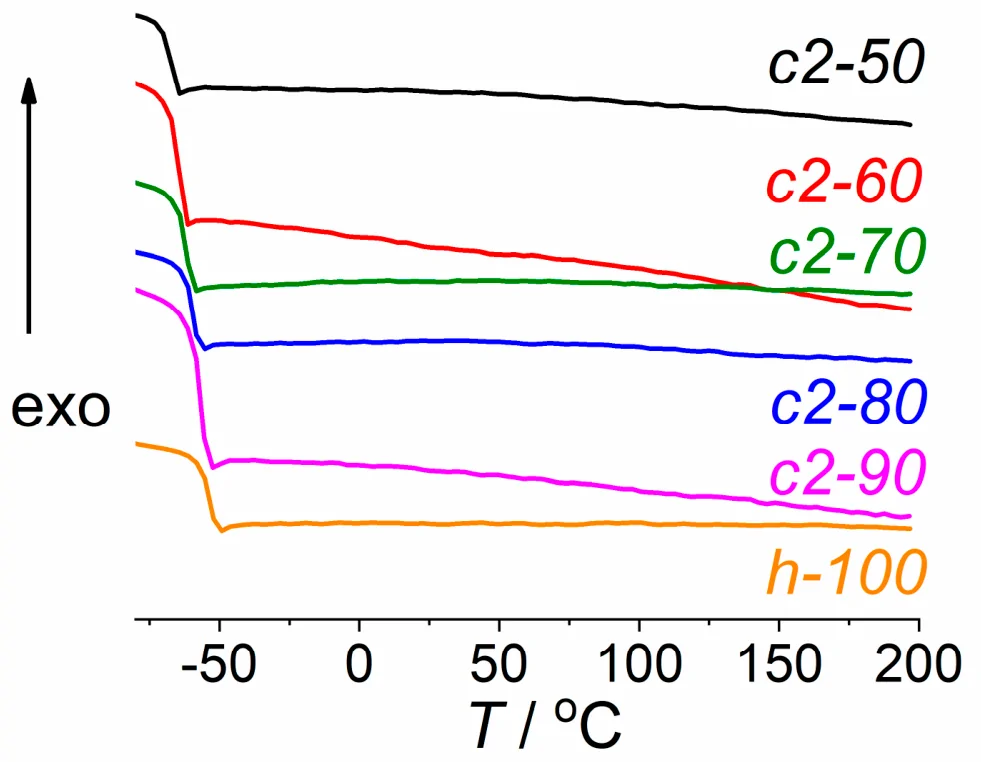
The differential scanning calorimetry curves of the copolymers obtained in the experimental series of *c2* (HDODA comonomer) and PDODT homopolymer (*h*-100).

**Figure 8:**
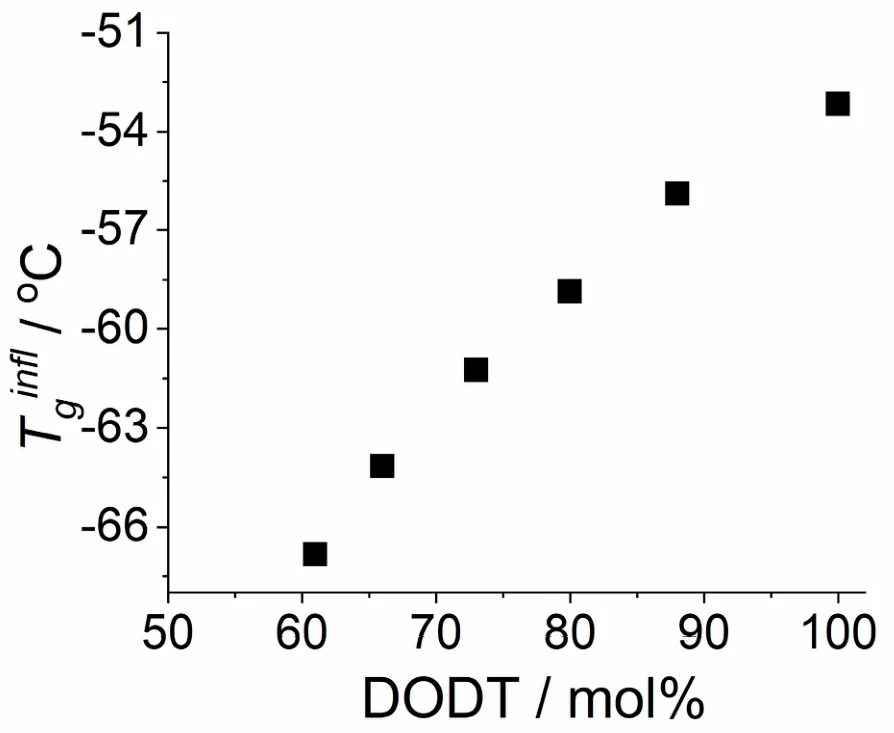
The glass transition temperatures as the function of the DODT content of the P(DODT-*co*-HDODA) copolymers obtained in the experimental series of *c2* (HDODA comonomer) and the PDODT homopolymer (*h*-100).

The DSC curves of the P(DODT-*co*-HDODA) copolymers show only one glass transition in the −100–+200 °C temperature range (Figure 7). However, in contrast to the P(DODT-*co*-PEGDA) copolymers, the glass transition temperatures (*T*_g_) of the HDODA-containing copolymers increase with the increasing DODT content, that is, it decreases with the increasing HDODA content (Figure 8). This is due to the hexamethylene segments of the HDODA monomeric units, which soften the macromolecules. This finding can be utilized to design such copolymers with predetermined *T*_g_ in a 15 °C range.

### 3.3. P(DODT-co-PEGDA) Copolymers by Sequential Monomer Addition

**Figure 9:**
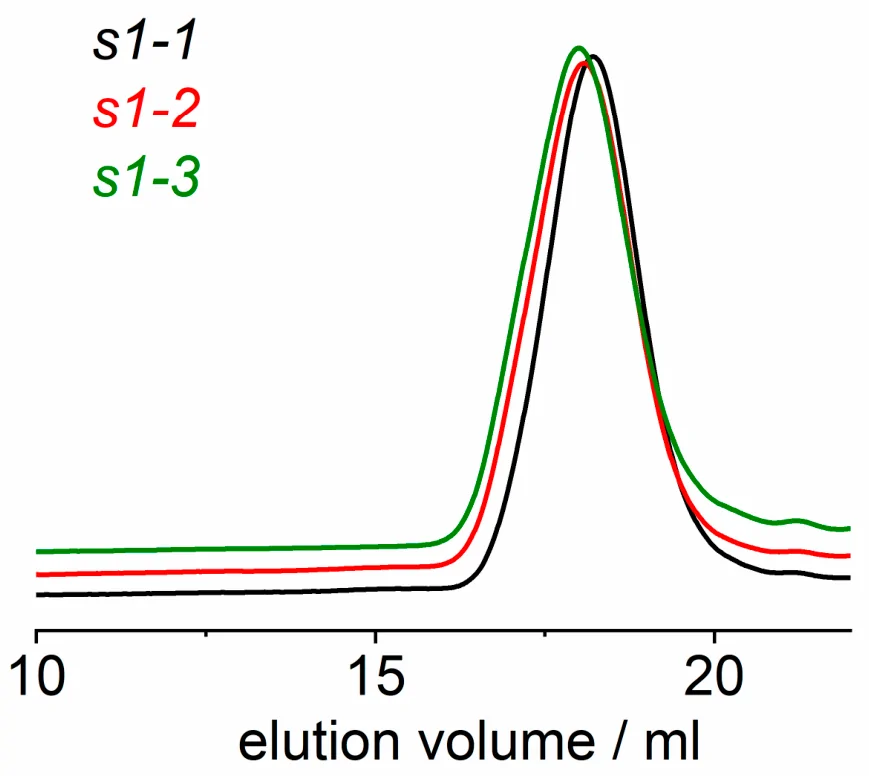
The GPC chromatograms of the P(DODT-*co*-PEGDA) copolymers obtained in the *s1* experimental series (simultaneous sequential addition of both DODT and PEGDA in stoichiometric ratio; the second number in the sample code stands for the number of the addition sequence).

The effect of sequential monomer and oxidant addition on the formation of P(DODT-*co*-PEGDA) copolymers was also tested. In the first experiment, after the preparation of a copolymer with 50:50 (mol%) DODT/PEGDA content in the feed, a sample was withdrawn, and a second addition of a monomer mixture also containing the same DODT/PEGDA ratio, followed by the addition of an equal molar amount of H_2_O_2_ solution, was performed. Both the ^1^H NMR spectra and the GPC chromatograms clearly show that neither the DODT content in the copolymers nor the *M*_n_ and dispersity change considerably in such a sequential addition of DODT-PEGDA mixtures with stoichiometric ratio (Figure 9 and Figure S3, Table 4). In addition, the yield of the second addition of the monomers is practically the same as in the first reaction sequence as shown in Table 4. These findings prove that the DODT and PEGDA, added in the second step, form mainly new copolymer chains, and only minor extension and/or coupling of the acrylate-telechelic copolymers, obtained in the first step, take place. After the third addition of the same monomer mixture, the occurrence of these phenomena is indicated as well.

**Figure 10:**
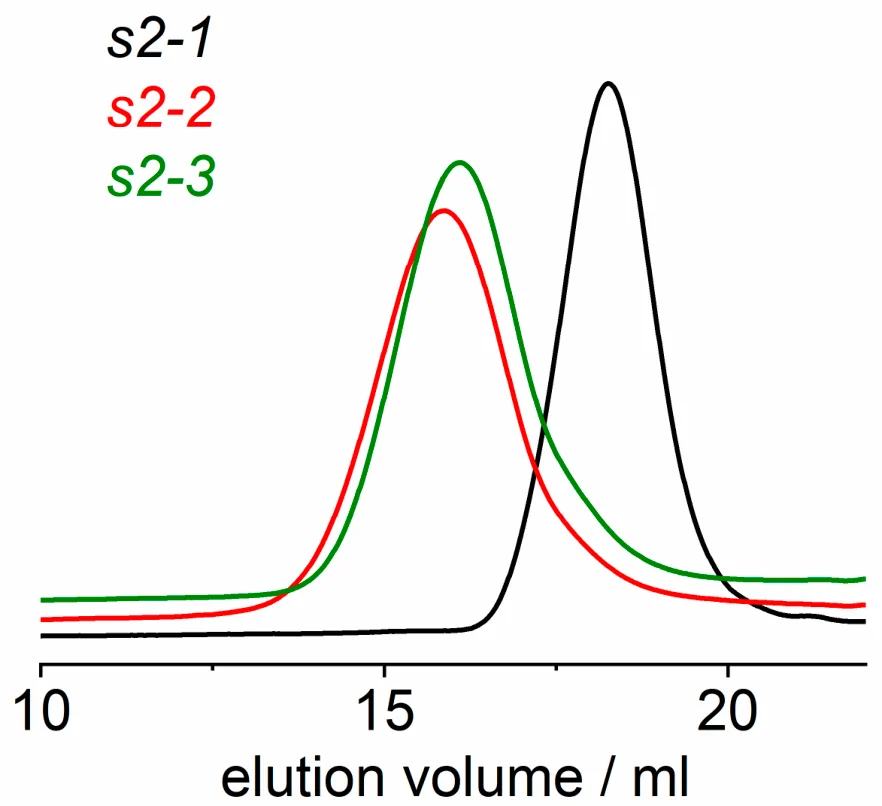
The GPC chromatograms of the P(DODT-*co*-PEGDA) copolymers obtained in the *s2* experimental series (sequential addition of only DODT and H_2_O_2_ oxidant; the second number in the sample code stands for the number of the addition sequence).

However, when the sequentially added monomer is only DODT, a considerable shift occurs in the gel permeation chromatograms (Figure 10 and Figure S4) indicating polymer chain extension, i.e., coupling of the previously formed acrylate-telechelic copolymers by the thiolates, formed by the thiol-base reaction, occurs due to the absence of monomeric acrylate groups. This is also confirmed by the PEGDA content in the copolymer, which is lower than before the sequential DODT addition (Table 4), on the one hand. On the other hand, neither the acrylic peaks nor the signals for free -SH groups appear in the ^1^H NMR spectrum of the copolymer formed after the subsequent charge of DODT and the oxidant. This indicates the formation of macrocyclic polymers as reported for PDODT homopolymers [32,33,34,35,36,37]. After the second charge of DODT and oxidant, the GPC chromatograph does not shift considerably to the higher molecular weights, indicating that the acrylate end groups are consumed in chain coupling during the previous DODT addition (sample *s2-3*). However, the DODT content is higher than before the second reagent addition, which is due to the polymerization of DODT also in this part of the experiment. The GPC curve and the somewhat decreased *M*_n_ of the copolymer formed after the second DODT and oxidant addition indicate the formation of some lower molecular weight polymers (Figure 10 and Figure S4, Table 4), which should be PDODT homooligomers.

### 3.4. Targeted Degradation of Copolymers by Reductive Scission and Hydrolysis with a Strong Base

**Figure 11:**
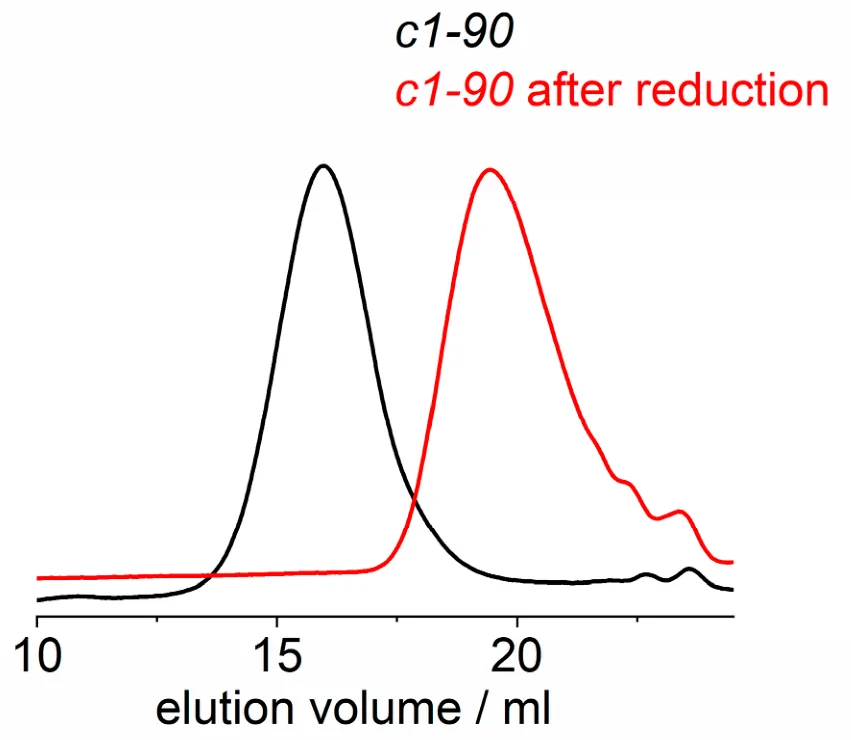
The GPC chromatograms of the *c1*-90 P(DODT-*co*-PEGDA) copolymer sample before and after reduction with 2-mercaptoethanol.

**Figure 12:**
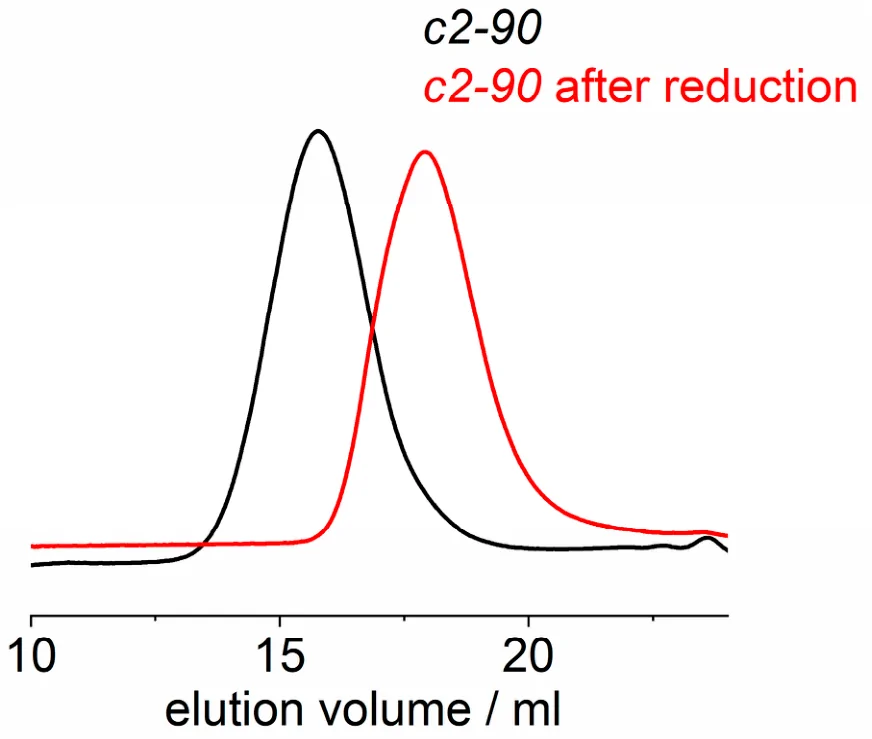
The GPC chromatograms of the *c2*-90 P(DODT-*co*-HDODA) copolymer before and after reduction with 2-mercaptoethanol.

As is well known, the disulfides (–S–S–) can undergo reductive scission in the presence of thiols. Therefore, reductive chain-breaking experiments were performed to investigate the redox sensitivity of the obtained copolymers. First, all the reduction experiments were performed with the copolymers having the highest DODT content, that is, the relatively highest disulfide linkages, in order to gain information about the presence of cleavable disulfide linkages in the P(DODT-*co*-PEGDA) copolymers. For the subsequent reduction studies by DTT, we deliberately switched to a copolymer containing 50 mol% DODT, as this composition is more promising for potential future applications. Thus, the PEGDA- and HDODA-containing copolymers with 90 mol% DODT content in the feed (*c1*-90 and *c2*-90 samples, respectively) were treated with 2-mercaptoethanol (2-ME). Figure 11, Figure 12, Figures S5 and S6 show that the GPC curves of the copolymers reacted with this thiol are shifted to the lower molecular weights in the case of both copolymers, proving the presence of the disulfide linkages in these copolymers, which undergo reductive chain scission, on the one hand. On the other hand, these results indicate the ability of these DODT-diacrylate copolymers for reductive decomposition.

Although the molecular weight data obtained by GPC can be considered as relative estimates due to the differences in the hydrodynamic volumes of the homopolymer standards used for calibration and that of the P(DODT-*co*-PEGDA) copolymers, the changes in the number average molecular weight (*M*_n_) can be used to obtain some information about the extent of degradation by the reductive treatment of the copolymers with a thiol. A sample calculation illustrating this approach is included in the Supplementary Materials. However, it must also be emphasized that the post-degradation *M*_n_ values are only approximate values, because it cannot be guaranteed that (i) all the low-molecular-weight fragments generated from the polymer are fully present, and that (ii) no reduction agent-derived species infiltrate the measured sample. Consequently, we refrain from presenting a detailed quantitative discussion of the extent of degradation here. Nevertheless, the results of the estimated values of the extent of degradation by treatment of the P(DODT-*co*-PEGDA) copolymers with a thiol are shown in Table S1. This estimation indicates relatively low values, which is presumably due to the uncertainties discussed above, the relatively low thiol/disulfide ratio and the hydrophobic nature of the copolymers. However, the significant shift in the GPC curves towards the lower molecular weights definitely proves the chain scission by the treatment with thiols, and thus convincingly indicates also the presence of the degradable disulfide linkages in these new copolymers, corroborating the results of the ^1^H NMR analysis of these novel copolymers.

**Figure 13:**
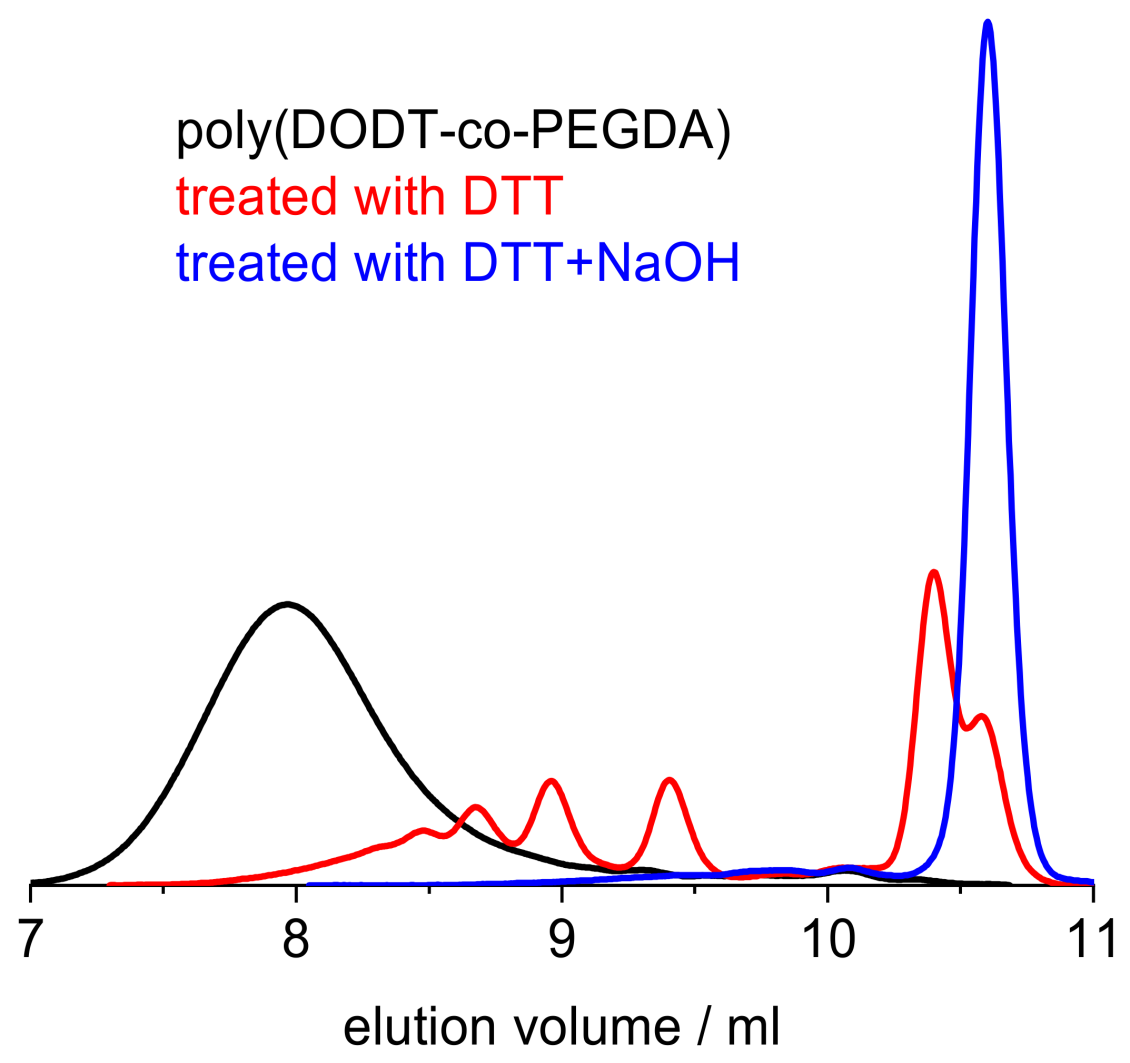
The GPC chromatograms of the P(DODT-*co*-PEGDA) copolymer obtained with 1:1 DODT/PEGDA ratio before and after reduction with dithiothreitol (DTT) followed by treatment with NaOH solution.

By treating the P(DODT-*co*-PEGDA) copolymer, obtained in the scale-up process by using 1:1 molar ratio of DODT and PEGDA in the feed, with dithiothreitol (DTT), a biochemically relevant thiol, the GPC curve of the copolymer is shifted to lower molecular weight, indicating the presence and thus the reductive scission of the disulfide linkages in this copolymer. Additional treatment of the reductively degraded copolymer by sodium hydroxide solution on the basis of results by Junkers et al. [45] for degrading poly(β-thioester)s leads to further degradation as shown by the additional shift of the GPC chromatogram to the lower molecular weight region as displayed in Figure 13 and Figure S7. This is corroborated by FTIR measurements as well as indicated by the data in Figure S8. The FTIR spectra clearly show that the carbonyl stretching band of the ester group at 1730 cm^−1^ completely disappears, and two new bands can be observed for the carboxylate and carboxylate hydroxyl groups at 1570 cm^−1^ and 1400 cm^−1^, respectively. These findings indicate that targeted treatment of the DODT-diacrylate copolymers, formed by simultaneous oxidative disulfide formation and thiol-ene Michael addition, by thiols and strong bases enables highly efficient predetermined degradation of these novel polymers.

## 4. Conclusions

Simultaneous oxidative disulfide formation by thiol–thiol coupling, using H_2_O_2_ as an oxidant, and thiol-ene Michael addition click reaction between a dithiol (DODT) and two diacrylates (PEGDA and HDODA) in the presence of a base (PMDETA), as a common catalyst for both processes, was successfully achieved to obtain copolymers consisting of disulfide and β-thioester linkages, for the first time to the best of our knowledge. This way, redox-sensible P(DODT-*co*-PEGDA) and P(DODT-*co*-HDODA) copolymers were synthesized with tunable copolymer contents under mild conditions, that is, in open air at room temperature and rather short reaction times of 10–15 min. By using stoichiometric DODT/PEGDA ratios, acrylate-telechelic copolymers are obtained directly without the need of any additional chain end-functionalization process. These novel bifunctional, acrylate-ended copolymers can be utilized as novel macromolecular building elements for obtaining a broad range of sulfur-containing polymer architectures, such as polymers with other functionalities, block copolymers, networks and gels, etc. DSC experiments showed that both types of copolymers have low *T*_g_s in the range of −53 °C and below, indicating elastomer-like behavior of the formed copolymers at room temperature. Treating the copolymers with thiols, such as 2-mercaptoethanol and dithiothreitol, led to scission of the disulfide units via a thiol–disulfide exchange redox reaction. This indicates that these kinds of copolymers can be utilized in dynamic thiol–disulfide processes for various purposes, on the one hand. On the other hand, successful hydrolysis of the β-thioester linkages was carried out with NaOH solution, leading to low-molecular-weight products. These findings indicate that combining these two degradation processes, that is, disulfide breaking by reduction and hydrolysis of the ester groups in these copolymers, enables us to fully degrade these novel P(DODT-*co*-diacrylate) copolymers. In sum, the novel robust, rapid and mild process, reported in this study, by utilizing the same base as catalyst for the simultaneous oxidative disulfide formation and thiol-ene Michael addition click reaction results in new degradable acrylate-telechelic sulfur-containing copolymers, which can be utilized in a large variety of advanced applications.

## Figures and Tables

**Table 1:** The DODT content in the feed and the resulting P(DODT-*co*-PEGDA)s obtained by copolymerization of DODT with PEGDA, the yield, the number average molecular weights (*M*_n_), the peak molecular weights at the maxima of the GPC curves (*M*_peak_) and dispersity (*Đ*) of the polymers (in the sample codes “1” means the experimental series number, while the second number stands for the DODT content in the feed).

Sample	DODT in the Feed (mol%)	DODT in the Copolymer ^a^ (mol%)	Yield (%)	*M*_n_ ^a^ by ^1^H NMR (kg/mol)	*M*_n_ ^b^ (kg/mol)	*M*_peak_ ^b^ (kg/mol)	*Đ* ^b^
*c1*-50	50	58	40	4.5	6.0	7.0	1.3
*c1*-60	60	65	63	10.9	9.3	12.0	1.6
*c1*-70	70	74	67	n.d. ^c^	17.9	31.0	2.0
*c1*-80	80	82	72	n.d. ^c^	27.8	44.9	2.0
*c1*-90	90	91	72	n.d. ^c^	49.2	78.5	2.3
*h*-100	100	100	59	n.d. ^c^	23.9	39.1	2.0

^a^ on the basis of the ^1^H NMR spectra; ^b^ on the basis of the GPC measurements; ^c^ not determined due to the absence of the olefinic proton’s signals.

**Table 2:** The solubility of the P(DODT-*co*-PEGDA) copolymers in various solvents.

Solvent	Solubility of P(DODT-*co*-PEGDA) Copolymer
Water	insoluble
Methanol	insoluble
Ethanol	insoluble
Toluene	soluble
Tetrahydrofuran	soluble
Methyl ethyl ketone	soluble
Acetonitrile	soluble
Chloroform	soluble
Dichloromethane	soluble
Dimethylformamide	soluble
*n*-Hexane	insoluble
Diethyl ether	insoluble

**Table 3:** The DODT content in the feed and the resulting P(DODT-*co*-HDODA)s obtained by copolymerization of DODT with HDODA, the yield, the number average molecular weights (*M*_n_), the peak molecular weights at the maxima of the GPC curves (*M*_peak_) and the dispersity (*Đ*) of the polymers (in the sample codes “2” means the experimental series number, while the second number stands for the DODT content in the feed).

Sample	DODT in the Feed (mol%)	DODT in the Copolymer ^a^ (mol%)	Yield (%)	*M*_n_ ^a^ by ^1^H NMR (kg/mol)	*M*_n_ ^b^ (kg/mol)	*M*_peak_ ^b^ (kg/mol)	*Đ* ^b^
*c2*-50	50	61	31	2.4	3.4	4.5	1.4
*c2*-60	60	66	54	3.8	4.7	6.1	1.5
*c2*-70	70	73	60	5.7	6.2	8.5	1.6
*c2*-80	80	80	69	14.6	13.2	25.1	1.8
*c2*-90	90	88	67	n.d. ^c^	38.4	57.0	2.0
*h*-100	100	100	59	n.d. ^c^	23.9	39.1	2.0

^a^ on the basis of the ^1^H NMR spectra; ^b^ on the basis of the GPC measurements; ^c^ not determined due to the absence of the olefinic proton’s signals.

**Table 4:** The compositions, the yield, the number average molecular weights (*M*_n_) and the dispersity (*Đ*) of the P(DODT-*co*-PEGDA) copolymers formed in the sequential monomer addition experiments (*s1* series: simultaneous sequential addition of both DODT and PEGDA in stoichiometric ratio; *s2* series: sequential addition of only DODT and H_2_O_2_ oxidant; the second number in the sample code stands for the number of the addition sequence).

Sample	DODT in the Feed (mol%)	DODT in the Copolymer ^a^ (mol%)	Yield (%)	*M*_n_ ^a^ by ^1^H NMR (kg/mol)	*M*_n_ ^b^ (kg/mol)	*Đ* ^b^
*s1-1*	50	57	48	5.2	6.9	1.3
*s1-2*	50	58	45	6.0	7.6	1.4
*s1-3*	50	58	45	5.9	7.9	1.4
*s2-1*	50	58	42	5.1	6.6	1.3
*s2-2*	75	67	57	n.d. ^c^	36.5	2.1
*s2-3*	87.5	78	26	n.d. ^c^	30.4	1.9

^a^ on the basis of the ^1^H NMR spectra; ^b^ on the basis of the GPC measurements; ^c^ not determined due to the absence of the olefinic proton’s signals.

## Data Availability

Additional data inquiries can be directed to the corresponding authors.

## Supplementary Materials

The following supporting information can be downloaded at https://www.mdpi.com/article/10.3390/polym18182211/s1, Figure S1. The molecular weight distribution curves of the P(DODT-*co*-PEGDA) copolymers obtained in the *c1* experimental series (see Table 1 for sample identification). Figure S2. The molecular weight distribution curves of the P(DODT-*co*-HDODA) copolymers obtained in the *c2* experimental series (see Table 3 for sample identification). Figure S3. The molecular weight distribution curves of the P(DODT-*co*-PEGDA) copolymers obtained in the *s1* experimental series (simultaneous sequential addition of both DODT and PEGDA in stoichiometric ratio; the second number in the sample code stands for the number of the addition sequence). Figure S4. The molecular weight distribution curves of the P(DODT-*co*-PEGDA) copolymers obtained in the *s2* experimental series (sequential addition of only DODT and H_2_O_2_ oxidant; the second number in the sample code stands for the number of the addition sequence). Figure S5. The molecular weight distribution curves of the *c1*-90 P(DODT-*co*-PEGDA) copolymer sample before and after reduction with 2-mercaptoethanol. Figure S6. The molecular weight distribution curves of the *c2*-90 P(DODT-*co*-HDODA) copolymer sample before and after reduction with 2-mercaptoethanol. Figure S7. The molecular weight distribution curves of the P(DODT-*co*-PEGDA) copolymers obtained with 1:1 DODT/PEGDA ratio before and after reduction with dithiothreitol (DTT), and by subsequent treatment with NaOH solution. Figure S8. The FTIR spectra of the P(DODT-*co*-PEGDA) copolymer sample before (top) and after treatment with 2-mercaptoethanol and NaOH solution (bottom). Table S1. The applied copolymer sample, the number average molecular weights (*M*_n_) and dispersity (*Đ*) before and after reduction with 2-mercaptoethanol, and the extent of the disulfide decomposition by reduction.
