# Supplementary material for: Degradable Acrylate-Telechelic Copolymers with Disulfide and β-Thioester Linkages by Simultaneous Thiol Oxidation and Thiol-Ene Michael Addition Click Reactions with the Same Base Catalyst
Source: Polymers (Basel). 2026 Sep 11;18(18):2211. doi: 10.3390/polym18182211 (PMC13610968; doi:10.3390/polym18182211)
Supplement: Supplementary file 1 [file polymers-18-02211-s001.zip › polymers-4497853-supplementary.pdf]

SUPPLEMENTARY MATERIALS

Degradable Acrylate-Telechelic Copolymers with Disulfide and β-Thioester Linkages by Simultaneous Thiol Oxidation and Thiol-Ene Michael Addition Click Reactions with the Same Base Catalyst

Ákos Szabó[1*], Aiman Aitkazina[1,2], Györgyi Szarka[1], Dóra Fecske[1], Anna Petróczy[1], Béla Iván[1*]

[1] Polymer Chemistry and Physics Research Group, Institute of Materials and Environmental Chemistry, HUN-REN Research Centre for Natural Sciences, Magyar tudósok krt. 2, H-1117 Budapest, Hungary

[2] Hevesy György Doctoral School of Chemistry, Institute of Chemistry, Faculty of Science, Eötvös Loránd University, Pázmány Péter sétány 1/A, H-1117 Budapest, Hungary

Correspondence: szabo.akos@ttk.hu (Á.Sz.), ivan.bela@ttk.hu (B.I.)

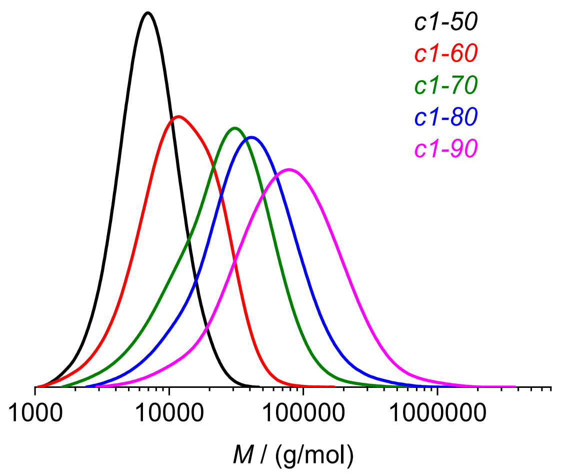

Figure S1. The molecular weight distribution curves of the P(DODT-*co*-PEGDA) copolymers obtained in the *c1* experimental series (see Table 1 for sample identification).

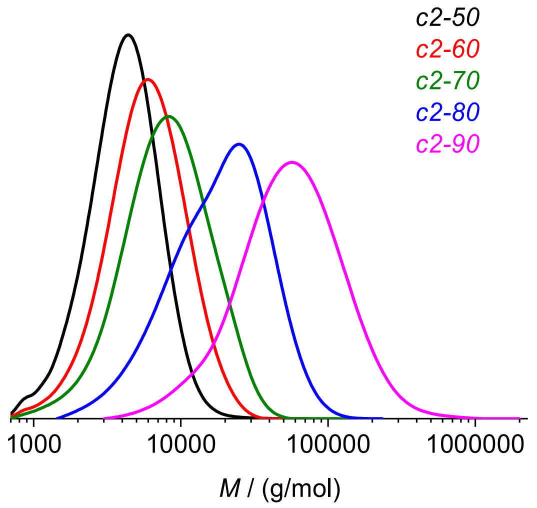

Figure S2. The molecular weight distribution curves of the P(DODT-*co*-HDODA) copolymers obtained in the *c2* experimental series (see Table 3 for sample identification).

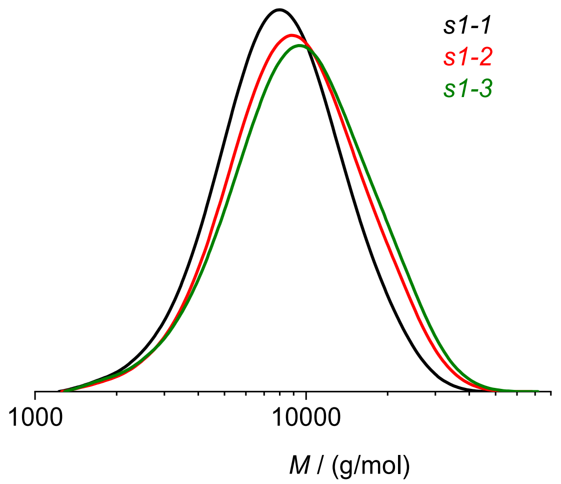

Figure S3. The molecular weight distribution curves of the P(DODT-*co*-PEGDA) copolymers obtained in the *s1* experimental series (simultaneous sequential addition of both DODT and PEGDA in stoichiometric ratio; the second number in the sample code stands for the number of the addition sequence).

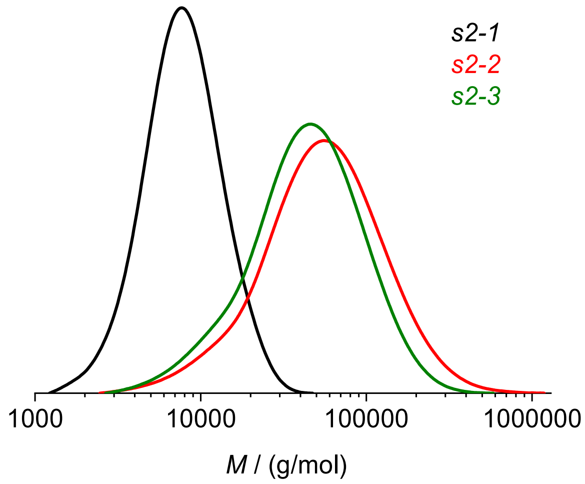

Figure S4. The molecular weight distribution curves of the P(DODT-*co*-PEGDA) copolymers obtained in the *s2* experimental series (sequential addition of only DODT and H_2O_2 oxidant; the second number in the sample code stands for the number of the addition sequence).

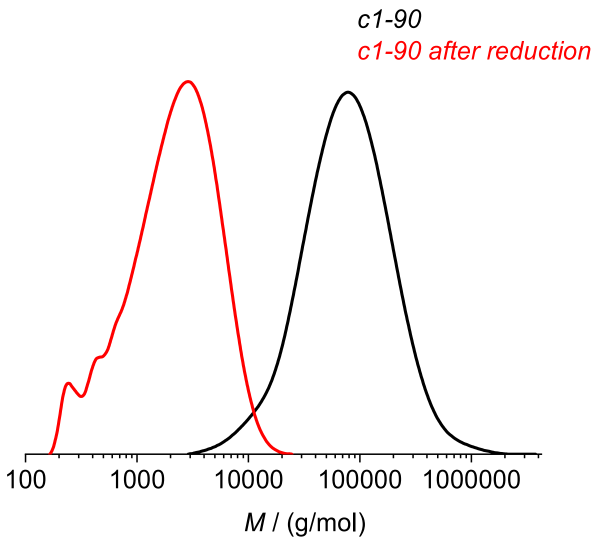

Figure S5. The molecular weight distribution curves of the *c1*-90 P(DODT-*co*-PEGDA) copolymer sample before and after reduction with 2-mercaptoethanol.

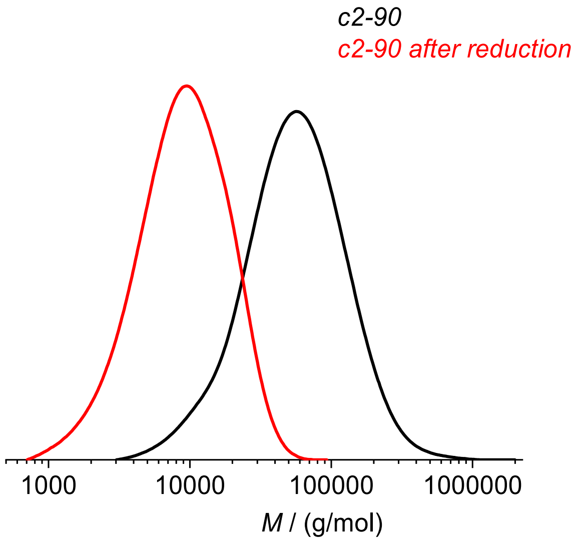

Figure S6. The molecular weight distribution curves of the *c*2-90 P(DODT-*co*-HDODA) copolymer sample before and after reduction with 2-mercaptoethanol.

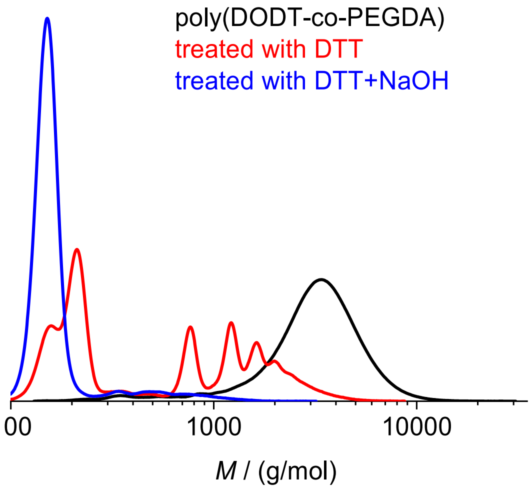

Figure S7. The molecular weight distribution curves of the P(DODT-*co*-PEGDA) copolymers obtained with 1:1 DODT/PEGDA ratio before and after reduction with dithiothreitol (DTT), and by subsequent treatment with NaOH solution.

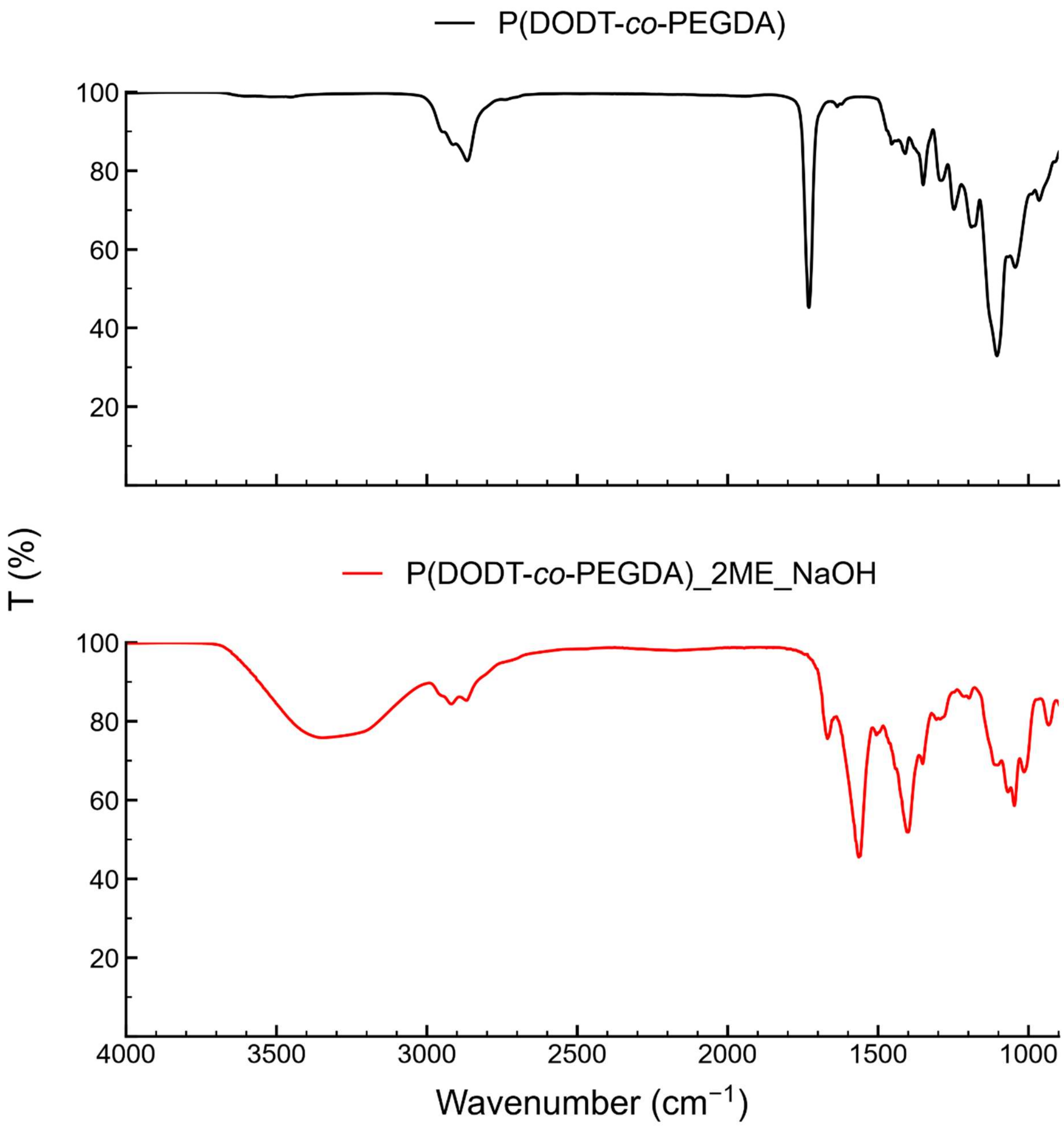

Figure S8. The FTIR spectra of the P(DODT-*co*-PEGDA) copolymer sample before (top) and after treatment with 2-mercaptoethanol and NaOH solution (bottom).

Table S1. The applied copolymer sample, the number average molecular weights (M_n) and dispersity ($Đ$) before and after reduction with 2-mercaptoethanol, and the extent of the disulfide decomposition by reduction.

**Sample**	**before reduction**		**after reduction**		**extent of the disulfide decomposition**
	**M_n (kg/mol)**	**$Đ$**	**M_n (kg/mol)**	**$Đ$**	
c1-90	49.2	2.3	1.3	2.2	0.18
c2-90	38.4	2.0	6.6	1.7	0.03

Calculation of the extent of the disulfide decomposition by reduction

The average molar mass of the monomer units in the copolymer (considering that DODT content is 91 mol%, see Table 1.):

$$M_{av}(\mathrm{mu}) = (1\text{-}0.91)\cdot 250+(1\text{-}0.91)\cdot 182+(0.91\text{-}(1\text{-}0.91))\cdot 180 = 168.48\ \mathrm{g/mol}$$

molar amount of disulfide groups in *m* g of copolymer:

$$n(\mathrm{S\text{-}S}) = m/168.48\cdot(0.91\text{-}(1\text{-}0.91))\ \mathrm{mol}$$

molar amount of reductively decomposed disulfide groups in *m* g of copolymer:

$$n(\mathrm{red.\ S\text{-}S}) = (m/1300)\text{-}(m/49200)\ \mathrm{mol}$$

their ratio is the extent of the disulfide decomposition by reduction:

$$n(\mathrm{red.\ S\text{-}S})/n(\mathrm{S\text{-}S}) = 0.18$$
